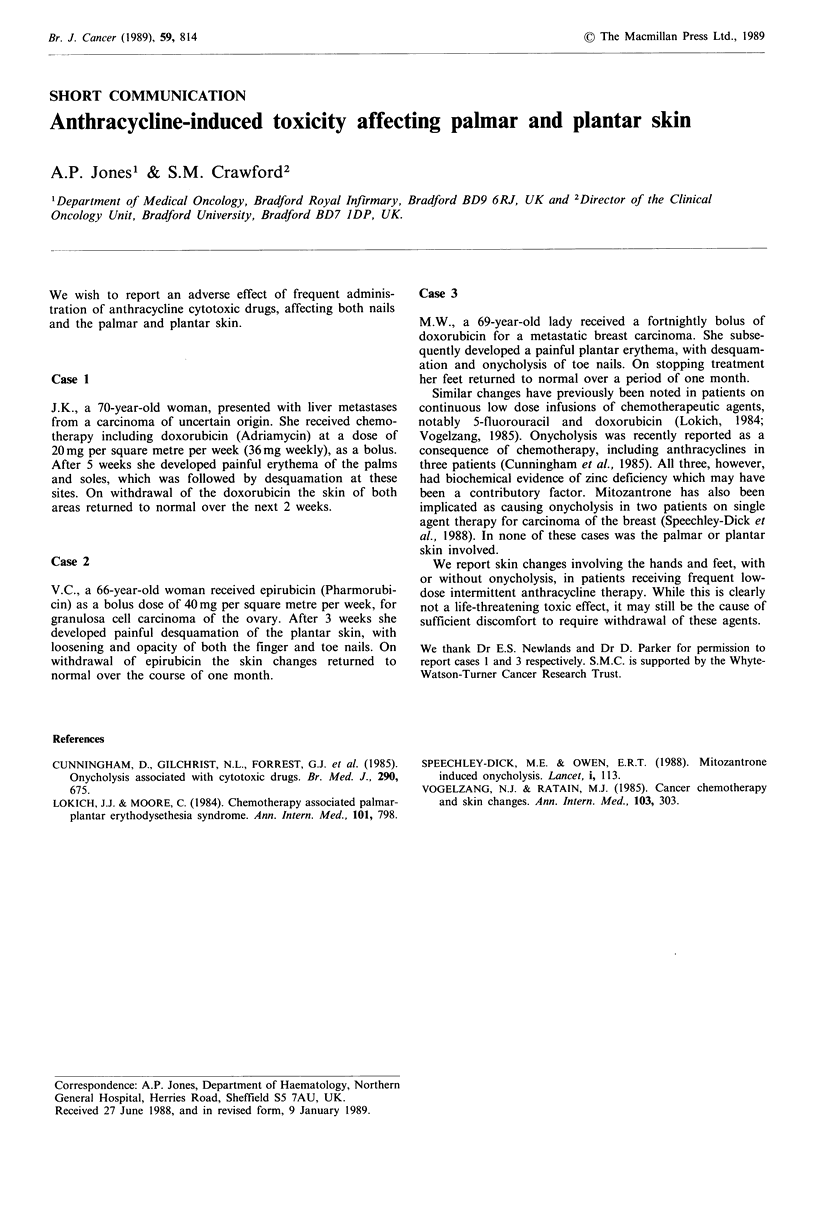# Anthracycline-induced toxicity affecting palmar and plantar skin.

**DOI:** 10.1038/bjc.1989.170

**Published:** 1989-05

**Authors:** A. P. Jones, S. M. Crawford

**Affiliations:** Department of Medical Oncology, Bradford Royal Infirmary, UK.


Br. J. Cancer (1989), 59, 814
© The Macmillan Press Ltd., 1989

SHORT COMMUNICATION

### Anthracycline-induced toxicity affecting palmar and plantar skin

A.P. Jones[1] & S.M. Crawford[2]

[1]*Department of Medical Oncology, Bradford Royal Infirmary, Bradford BD9 6RJ, UK and* [2]*Director of the Clinical Oncology Unit, Bradford University, Bradford BD7 1DP, UK.*

We wish to report an adverse effect of frequent administration of anthracycline cytotoxic drugs, affecting both nails and the palmar and plantar skin.

### Case 1

J.K., a 70-year-old woman, presented with liver metastases from a carcinoma of uncertain origin. She received chemotherapy including doxorubicin (Adriamycin) at a dose of 20 mg per square metre per week (36 mg weekly), as a bolus. After 5 weeks she developed painful erythema of the palms and soles, which was followed by desquamation at these sites. On withdrawal of the doxorubicin the skin of both areas returned to normal over the next 2 weeks.

### Case 2

V.C., a 66-year-old woman received epirubicin (Pharmorubicin) as a bolus dose of 40 mg per square metre per week, for granulosa cell carcinoma of the ovary. After 3 weeks she developed painful desquamation of the plantar skin, with loosening and opacity of both the finger and toe nails. On withdrawal of epirubicin the skin changes returned to normal over the course of one month.

### Case 3

M.W., a 69-year-old lady received a fortnightly bolus of doxorubicin for a metastatic breast carcinoma. She subsequently developed a painful plantar erythema, with desquamation and onycholysis of toe nails. On stopping treatment her feet returned to normal over a period of one month.

Similar changes have previously been noted in patients on continuous low dose infusions of chemotherapeutic agents, notably 5-fluorouracil and doxorubicin (Lokich, 1984; Vogelzang, 1985). Onycholysis was recently reported as a consequence of chemotherapy, including anthracyclines in three patients (Cunningham *et al.*, 1985). All three, however, had biochemical evidence of zinc deficiency which may have been a contributory factor. Mitozantrone has also been implicated as causing onycholysis in two patients on single agent therapy for carcinoma of the breast (Speechley-Dick *et al.*, 1988). In none of these cases was the palmar or plantar skin involved.

We report skin changes involving the hands and feet, with or without onycholysis, in patients receiving frequent low-dose intermittent anthracycline therapy. While this is clearly not a life-threatening toxic effect, it may still be the cause of sufficient discomfort to require withdrawal of these agents.

We thank Dr E.S. Newlands and Dr D. Parker for permission to report cases 1 and 3 respectively. S.M.C. is supported by the Whyte-Watson-Turner Cancer Research Trust.

### References

CUNNINGHAM, D., GILCHRIST, N.L., FORREST, G.J. *et al.* (1985). Onycholysis associated with cytotoxic drugs. *Br. Med. J.*, **290**, 675.

LOKICH, J.J. & MOORE, C. (1984). Chemotherapy associated palmar-plantar erythodysethesia syndrome. *Ann. Intern. Med.*, **101**, 798.

SPEECHLEY-DICK, M.E. & OWEN, E.R.T. (1988). Mitozantrone induced onycholysis. *Lancet*, **i**, 113.

VOGELZANG, N.J. & RATAIN, M.J. (1985). Cancer chemotherapy and skin changes. *Ann. Intern. Med.*, **103**, 303.

Correspondence: A.P. Jones, Department of Haematology, Northern General Hospital, Herries Road, Sheffield S5 7AU, UK.
Received 27 June 1988, and in revised form, 9 January 1989.